# Growth and Physiological Responses of Temperate Pasture Species to Consecutive Heat and Drought Stresses

**DOI:** 10.3390/plants8070227

**Published:** 2019-07-16

**Authors:** Ruchika S. Perera, Brendan R. Cullen, Richard J. Eckard

**Affiliations:** Faculty of Veterinary and Agricultural Sciences, University of Melbourne, Melbourne, VIC 3010, Australia

**Keywords:** perennial pastures, combined heat and drought stress, membrane permeability, maximum photochemical efficiency of PS II, acclimation

## Abstract

Heat and drought are two major limiting factors for perennial pasture production in south eastern Australia. Although previous studies have focused on the effects of prolonged heat and drought stresses on pasture growth and physiology, the effects of short term recurring combined heat and drought stresses and the recovery from them have not been studied in detail. A controlled environment experiment was conducted to investigate the growth and physiological responses of perennial ryegrass (*Lolium perenne* L.), cocksfoot (*Dactylis glomerata* L.), tall fescue (*Festuca arundinacea* Schreb.) and chicory (*Cichorium intybus* L.) plants exposed to two consecutive seven day heat (control = 25/15 °C day/night; moderate = 30/20 °C day/night and severe = 35/30 °C day/night) and/or drought stresses each followed by a seven day recovery period. During the first moderate and severe heat and drought treatments, maximum photochemical efficiency of photosystem II (Fv/Fm), cell membrane permeability and relative leaf water content decreased in chicory and tall fescue compared to perennial ryegrass and cocksfoot. However, during the second moderate heat and drought treatment, all species showed less reduction in the same parameters suggesting that these species acclimated to consecutive moderate heat and drought stresses. Chicory was the only species that was not affected by the second severe heat and drought stress while physiological parameters of all grass species were reduced closer to minimum values. Irrigation mitigated the negative effects of heat stress by cooling the canopies 1–3 °C below air temperatures with the most cooling observed in chicory. All the species exposed to moderate heat and drought were fully recovered and those exposed to severe heat and drought recovered partially at the end of the experiment. These findings suggest that chicory may be a potential species for areas subject to frequent heat and drought stress.

## 1. Introduction

Global mean temperature is increasing at a rate of 0.2 °C per decade and projected to increase by 1.5 °C above pre-industrial levels (1850–1900) by 2050 if global warming continues at the current rate [1]. In conjunction with global temperature rise, mean temperature in Australia has increased by about 1 °C since the start of the 20th century [2]. At the same time, the frequency, magnitude and duration of extreme climate events such as heat waves and droughts are also increasing [2]. These climatic conditions are highly likely to challenge the production and persistence of perennial pastures in south eastern (SE) Australia, mainly in summer months (December–February). This in turn affects the profitability of the livestock industry since home grown pasture is the cheapest source of feed for livestock [3]. 

Heat stress is defined as a state where the temperatures are hot enough to cause impairment of physiology, metabolism and productivity of plants [4], where the impacts are mainly dependent on the duration and severity of the stress [5]. Drought stress is a period of sub-optimal water supply to plants that reduces water potential, turgor pressure and subsequently inhibits normal plant functions [6]. During prolonged severe drought, plants could be irreversibly damaged [7]. Heat and drought stress can act individually or in combination. Combinations of heat and drought stresses often cause more detrimental impacts on plants than individual stresses, such that when temperature increases, vapor pressure deficit rises allowing more transpiration per unit of CO_2_ uptake. This in turn reduces the water use efficiency [8] and increases the rate of water deficit stress development in plants. During water deficit stress, plant water status (water potential or relative water content) decreases affecting turgor pressure [6]. The first response to drought stress in plants is the reduction of leaf expansion. Since herbage mass is the primary target of pasture production, decreasing leaf expansion growth directly affects the forage dry matter production [9]. In SE Australia, rainfed perennial ryegrass pasture production in summer months is often as low as 5–10% of the annual yields, because of the suboptimal water availability and high temperature [10]. Reduction of leaf elongation rates due to water deficit stress has been widely reported for cool season temperate pasture species such as tall fescue and cocksfoot [11], perennial ryegrass [12,13] and Italian ryegrass [14]. In addition to that, water deficit largely impacts on stomatal opening and CO_2_ diffusion into the leaves, affecting photosynthetic carbon fixation [15,16,17,18,19].

The downregulation of photosynthesis due to heat stress occurs through reduction in Rubisco activity and impairment of photochemistry [20,21]. Optimal temperature for shoot growth of temperate cool season grasses range between 15–23 °C [22,23] and reduction in photosynthesis and growth beyond this range has been reported in several studies. It was found that photosynthesis of Kentucky bluegrass decreased at temperatures above 25 °C [24]. For tall fescue, reduction in photosynthesis has been reported at much higher temperatures (30 °C) [25,26,27].

Photosystem (PS) II is the most heat labile component in the photosynthesis process and is responsible for the impairment of photochemistry under heat stress [28,29,30]. Chlorophyll fluorescence has been developed as a non-destructive measurement to analyze abiotic stresses like heat and drought [31]. Measurement of fluorescence in dark adapted leaves provides an estimate of the maximum photochemical efficiency of PS II (Fv/Fm = variable fluorescence/maximum fluorescence) and gives important information on how much incoming light is used for the photochemistry. Fv/Fm in healthy leaves under optimum conditions is around 0.83 [32,33,34,35,36]. However, reductions of Fv/Fm have been reported under heat and drought stresses for pastures [24,25,32,37]. 

Downstream effects of heat stress include production of reactive oxygen species (ROS) [38] and peroxidation of membrane lipids. As a result, cell membranes lose their integrity and electrolyte leakage occurs [39]. Increased production of ROS like hydrogen peroxide and superoxide in tall fescue [40] and high lipid peroxidation in tall fescue and perennial ryegrass [25,41] have been observed under high temperature stress. However, variations exist among and within species with respect to their ability for thermotolerance. For instance, tall fescue and cocksfoot were reported to have higher membrane thermotolerance than perennial ryegrass [11,41,42]. 

Plants often evolve strategies to modify their responses to abiotic stresses using the previous stress memory, referred to as a priming effect [42]. Many studies have investigated the modified plant responses by exposing plants to non-lethal heat treatments [41,43]. Thermotolerance is acquired through reorganizing the lipid compositions of cell membranes [44] and modifying biochemical processes [45]. It has been reported that heat acclimated tall fescue and perennial ryegrass showed decreased water loss, membrane damage and lipid peroxidation relative to non-heat acclimated plants [41].

Growth and physiological responses of temperate pastures under prolonged heat and drought stresses have been studied previously [32,33,46]. However, heat and drought are likely to increase with the increased frequency of extreme climate events due to climate change [47]. Physiological responses of temperate grasses to recurring heat and drought stresses, and their ability to acclimate to the previous stresses have not been studied in detail. Therefore, the objectives of this study were to 1. investigate the growth and physiological responses and recovery potential of four temperate perennial pasture species that are commonly grown pasture species in SE Australia to consecutive heat and/or drought stress alone or in combination and 2. to identify pasture species more tolerant to recurring heat and drought stresses. To test these objectives, an experiment was conducted where plants were exposed to consecutive moderate (30/25 °C, day/night) and severe (35/30 °C, day/night) heat stresses under well-watered and drought conditions, with a one-week recovery period in between. 

## 2. Results

### 2.1. Relative Soil Water Content (RSWC)

**Figure 1:**
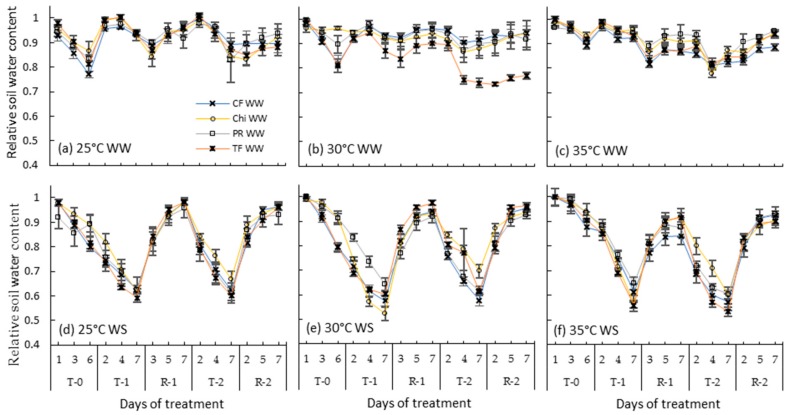
Relative soil water content of pots containing perennial ryegrass (PR), cocksfoot (CF), tall fescue (TF) and chicory (Chi) during consecutive heat and drought treatments and subsequent recovery periods. (**a**), (**b**) and (**c**) represent well-watered (WW) plants grown at control, moderate and severe heat stresses, respectively, while (**d**), (**e**) and (**f**) represent corresponding water stressed (WS) plants. T-0 denotes pre-treatment, T-1 and T-2 denote treatments and R-1 and R-2 denote recovery periods. Mean values (*n* = 5) are provided with error bars representing ± standard error

There was little difference in RSWC in well-watered treatments (Figure 1a–c) but it declined from the initial level of ~1 to about 0.6 on average at the end of the first combined heat and drought treatments. There was a difference between chicory and the grasses during the second drought treatment whereby chicory had higher RSWC than grasses at every temperature (Figure 1d–f). Soil water was restored in all the species during the recovery phases and the species differences were seldom significant at the end of each recovery phase (Table 1).

### 2.2. Maximum Photochemical Efficiency of Photosystem II (Fv/Fm)

**Figure 2:**
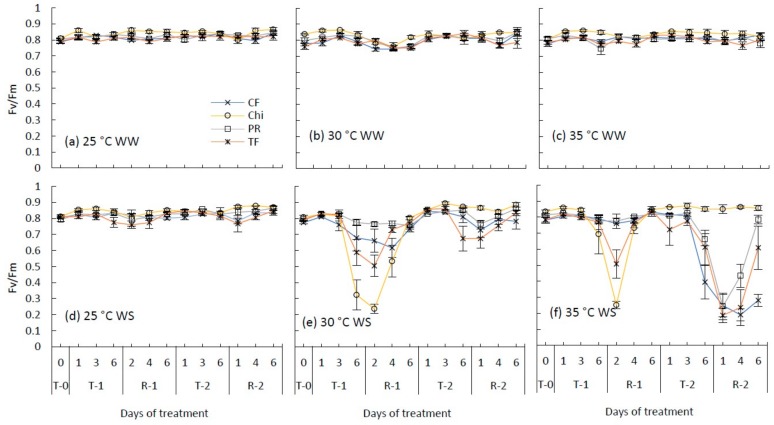
Maximum photochemical efficiency of photosystem II (Fv/Fm) during consecutive temperature and drought treatments and subsequent recovery of cocksfoot (CF), chicory (Chi), perennial ryegrass (PR) and tall fescue (TF). (**a**), (**b**) and (**c**) represent well-watered (WW) plants grown at control, moderate and severe heat stress, respectively, while (**d**), (**e**) and (**f**) represent corresponding water stressed (WS) plants. T-0 denotes pre-treatment, T-1 and T-2 denote treatments and R-1 and R-2 denote recovery periods. Mean (*n* = 3–5) values are provided with error bars representing ± standard error.

There was little difference in Fv/Fm ratio between species under well-watered conditions however, chicory had a higher Fv/Fm ratio compared to the other three grass species (Figure 2). Significant interactions between species, temperature and water status were observed at the end of the first treatment period then again after day 6 of the second treatment (Table 2). During the first moderate heat and drought, the maximum reduction of the Fv/Fm was observed in chicory (72% of the control), followed by tall fescue (38%), cocksfoot (17%) and perennial ryegrass (7%) on day 2 of recovery 1 (Figure 2). Similar patterns were observed for the first severe heat and drought treatment. However, reduction of Fv/Fm during the first severe heat and drought stress treatment was slower in pasture species than the first moderate stress. This may have been due to the high relative humidity (RH%) (>80%) observed during the first two days in the severe heat and stress-imposed growth chamber. High RH% may have reduced the rate of water loss from plants via transpiration and maintained plant functions longer into the stress.

During the second moderate heat and drought stress treatment, Fv/Fm was higher in all species compared to the first moderate heat and drought treatment (Figure 2e). For example, tall fescue had a reduction of 38% after the first treatment and it was only 17% after the second treatment. In contrast, in the second severe heat and drought treatment, the Fv/Fm of all species except chicory declined more significantly and extensively than the first severe heat and drought stress treatment. For example, perennial ryegrass had a reduction of 5% at the end of the first severe treatment compared to 71% at the end of the second severe treatment. During the recovery phase, perennial ryegrass almost fully recovered (94%) and cocksfoot only partially recovered (33%).

**Figure 3:**
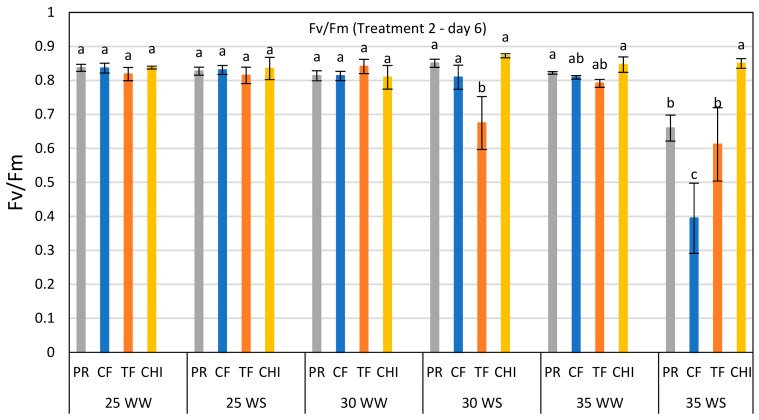
Comparison of Fv/Fm ratio of perennial ryegrass (PR), cocksfoot (CF), tall fescue (TF) and chicory (CHI) on day 6 of the second heat and water stress treatment. Each bar represents mean (*n* = 3–5) Fv/Fm value with ±SE. Significant differences are shown by different letters. The values in this bar graph can also be read from the day 6 of T-2 in each graph of Figure 2.

The significant interaction of the temperature × water status × species on day 6 of treatment 2 in Fv/Fm is shown in Figure 3. It showed that water stressed perennial ryegrass, cocksfoot and tall fescue plants in the severe temperature treatment (35 °C) had lower Fv/Fm values than the well-watered control. In contrast, chicory maintained a higher Fv/Fm value (similar to non-stressed control = 0.85). Chicory was the only species that did not show any reduction throughout the second heat and drought stress and recovery after stress, indicating no heat induced damage to chicory. 

### 2.3. Cell Membrane Permeability

**Figure 4:**
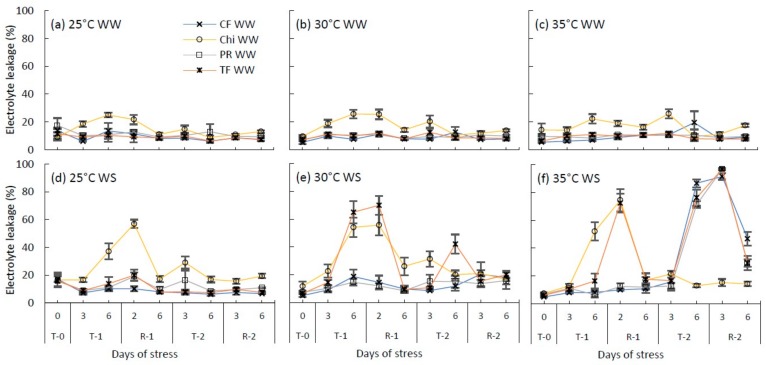
Changes to the cell membrane permeability (measured using electrolyte leakage %) of cocksfoot (CF), chicory (Chi), perennial ryegrass (PR) and tall fescue (TF) during the consecutive temperature and water treatments and subsequent recovery phases. (**a**), (**b**) and (**c**) represent well-watered (WW) plants grown at control, moderate and severe heat stress, respectively, while (**d**), (**e**) and (**f**) represent corresponding water stressed plants (WS). T-0 denotes pre-treatment, T-1 and T-2 denote treatments and R-1 and R-2 denote recovery periods. Mean values (*n* = 3–5) are provided with error bars representing ± standard error.

Electrolyte leakage (EL%), an indicator of cell membrane damage, did not increase under well-watered conditions (Figure 4a–c). However, chicory maintained slightly higher EL% (~16% on average) compared to other species (~10%). At the control temperature, drought stressed plants of chicory showed a gradual increase of EL% up to 60% in the first treatment but there were no marked variations in the EL% in any species during the second drought stress treatment at 25 °C. 

During the first moderate heat and drought stress treatment, chicory and tall fescue showed significant membrane damage up to 56% and 70%, respectively, extending the effect to the second day of the recovery phase. Similarly, during the first severe heat and drought stress treatment, the EL% of chicory and tall fescue increased significantly up to 74% and 72% but did not increase in perennial ryegrass or cocksfoot.

During the second moderate heat and drought treatment, electrolyte leakage of the tall fescue and chicory was lower compared to the first treatment. In contrast, cell membranes of all the grass species were significantly damaged (>90%) during the second combined severe heat and drought stress treatment and the effect extended to the recovery phase, but chicory was not affected. Chicory produced new leaf after the first stress period and these new leaves did not show EL above 15% during the second treatment period, indicating no heat induced membrane damage. At the end of the recovery phase, the grass species were partially recovered (Table 3), reporting EL% of 47% in cocksfoot and 30% in perennial ryegrass and tall fescue species.

### 2.4. Leaf Elongation Rates

**Figure 5:**
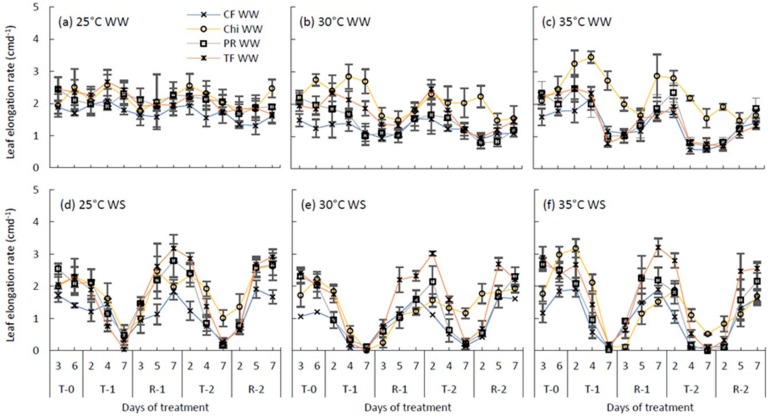
Leaf elongation rates of perennial ryegrass (PR) cocksfoot (CF), tall fescue (TF) and chicory (Chi) measured as cmd^−1^ during the consecutive temperature and drought treatments and subsequent recovery phases. (**a**), (**b**) and (**c**) represent well-watered (WW) plants grown at control, moderate and severe temperatures, respectively, while (**d**), (**e**) and (**f**) represent corresponding water stressed (WS) plants. T-0 denotes pre-treatment, T-1 and T-2 denote treatments and R-1 and R-2 denote recovery periods. Mean values (*n* = 3–5) are provided with the error bars representing the ± standard error.

Leaf elongation rates showed variations between species and ranged from 1.7 cmd^−1^ in cocksfoot to 2.2 cmd^−1^ in chicory on average under control temperature and well-water treatment (Figure 5a). Chicory showed a transient increase in leaf elongation rates under well-watered conditions soon after the temperature was increased (Figure 5b,c). However, heat stress alone decreased the leaf elongation rates of other grass species, particularly at the severe heat stress treatment (Figure 5c). For example, during the second severe heat treatment, leaf elongation rate of cocksfoot, perennial ryegrass and tall fescue decreased by 68%, 65% and 55%, respectively, compared to the control. Under water stress treatment (control temperature), leaf elongation rates of all the species decreased substantially and fluctuated around 0.3–0.5 cmd^−1^. At the end of both moderate and severe heat stresses, leaf elongation of water stressed plants stopped completely. In contrast, leaf elongation rate of chicory during the second combined moderate/severe heat and drought stresses did not reach zero but remained less than well-watered treatments. At the end of the recovery period, all plant species subjected to each temperature and drought treatment restored their leaf elongation rates to the control levels (Table 4) and tall fescue increased even above the control level.

### 2.5. Relative Leaf Water Content (RLWC)

**Figure 6:**
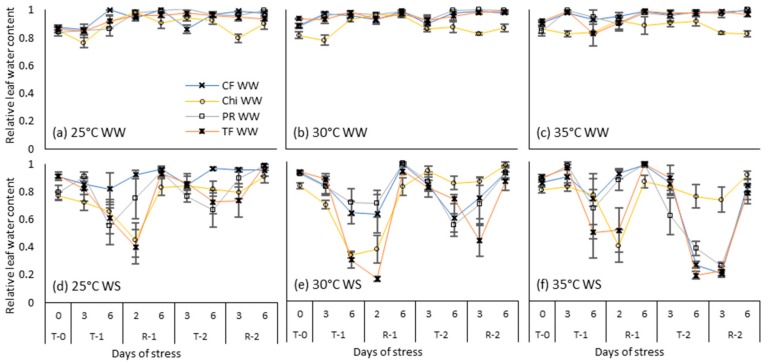
Relative leaf water content during consecutive temperature and drought treatments and subsequent recovery phases of cocksfoot (CF), chicory (Chi), perennial ryegrass (PR) and tall fescue (TF). (**a**), (**b**) and (**c**) represent well-watered (WW) plants grown at control, moderate and severe temperatures respectively while (**d**), (**e**) and (**f**) represent corresponding water stressed (WS) plants. T-0 denotes pre-treatment, T-1 and T-2 denote treatments and R-1 and R-2 denote recovery periods. Mean values (*n* = 3–5) are provided with error bars representing ± standard error.

Well-watered plants maintained higher RLWC >0.8 at all temperature treatments (Figure 6a–c). However, chicory displayed lower RLWC than grasses from the start of the experiment. At the end of the first treatment, the RLWC of chicory and tall fescue declined significantly to below control levels under all the temperature and drought stressed treatments with the lowest RLWC of 0.16 observed in tall fescue at moderate heat stress. During the second treatment, reduction in the RLWC in control and the moderate temperatures was much lower than the first treatment and chicory maintained a RLWC of ~0.8 which was closer to its non-stressed control value. The largest reductions of RLWC were observed at the end of the second severe heat and drought stressed treatment where the RLWC of all the grass species declined to ~0.2 but chicory maintained a greater RLWC of >0.7 All the grass species were almost recovered at the end of the second recovery period (Table 5), restoring the RLWC > 0.75.

### 2.6. Canopy Temperature Depression (CTD)

**Figure 7:**
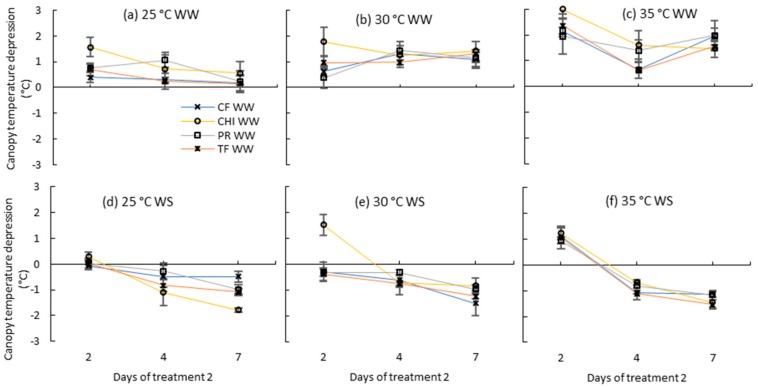
Canopy temperature depression (Ta-Tc) of perennial ryegrass (PR), cocksfoot CF), tall fescue (TF) and chicory (CHI) during day 2, 4 and 7 of the second treatment period. (**a**), (**b**) and (**c**) represent well-watered (WW) plants grown at control, moderate and severe temperatures, respectively, while (**d**), (**e**) and (**f**) represent corresponding water stressed (WS) plants. Means are shown (*n* = 3 or 5) with error bars representing ± standard error.

CTD of well-watered plants during treatment 2 were often positive at all temperature treatments (Figure 7a–c) indicating that the canopy was cooler than air temperature. Chicory showed the highest CTD at day 2 with cooling of 1.6 °C, 1.8 °C and 3 °C at control, moderate and severe heat stresses, respectively. In contrast all water stressed plants at day 4 and 7 were hotter than well-watered plants. At day 2, the canopy temperature of control and moderate heat stress were closer to air temperature, but at 35 °C, canopies were still 1 °C cooler. Furthermore, temperature and watering interaction was significant at day 7 (Table 6) indicating that the difference between well-watered and water stressed plants became higher when the severity of the high temperature stress increased. 

### 2.7. Top Dry Weight (Green, Senesced), Root Dry Weight and Root Length

**Figure 8:**
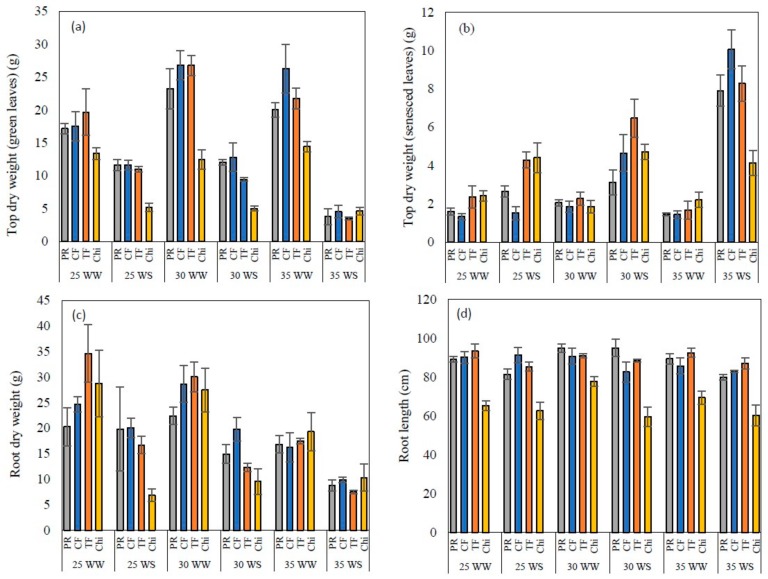
Top dry weight of green (**a**) and senesced leaves (**b**), root dry weight (**c**) and root length (**d**) of perennial ryegrass (PR), cocksfoot CF), tall fescue (TF) and chicory (Chi) measured at the end of the experiment. Mean values *n* = 5 are shown with the error bars representing standard error.

Dry matter yields of green leaves and roots showed clear differences between well-watered and water stressed plants and between species at the end of the experiment (Table 7, Figure 8a,c). Dry weight of green leaves and roots were greater in well-watered plants compared to water stressed plants. Further, higher temperatures (moderate and severe heat stress) increased the dry weight of green leaves compared to the control under well-watered conditions, but water stress counteracted this positive response. In contrast, the weight of the senesced leaves was greater in the water stressed plants and the highest senesced leaf weight was observed in combined severe heat and drought stress treatment. Even though root dry weight declined with increasing temperature and drought stress, root lengths did not show much variations across treatments. All grass species had root lengths of ~80 cm and chicory fluctuated around 65 cm. 

## 3. Discussion

In this study, all the species acclimated to a combination of moderate heat and drought, but only chicory acclimated to the combination of severe heat and drought stresses. Chicory not only tolerated consecutive severe heat and drought stress but also maintained growth as indicated by greater Fv/Fm, leaf elongation rates, RLWC and cell membrane stability at the end of the second severe heat and drought stress. This superior heat and drought tolerance of chicory is consistent with previous findings that showed chicory survived under supraoptimal temperatures (38/25 °C, day/night) and drought stresses for 18 days while other grass species including perennial ryegrass and cocksfoot died after 12 days of the same stress [33,48]. However, it is also important to note that the grasses did recover (partially) at the end of the experiment in the present study, suggesting that the consecutive heat and drought stresses of this magnitude were not severe enough to challenge the survival of the tested temperate grass species.

### 3.1. Individual Stresses 

#### 3.1.1. Effects of Heat Stress

Leaf elongation rates of all species were significantly affected by the severe heat stress in this study, confirming that growth rate of the youngest leaf is sensitive to the temperature changes in its growing environment [49]. A rapid decline of shoot growth of perennial ryegrass under high temperatures above 30 °C with complete cessation of growth at temperatures above 35 °C has been observed previously [50]. However, other physiological parameters such as Fv/Fm, EL% and RLWC of the four species were not affected by seven day moderate or severe heat stresses alone. A previous study has reported similar results that most of the cool season pastures in SE Australia (including pasture species used in this study) maintained Fv/Fm values near 0.83 (similar to unstressed plants) during heat stress treatment of 38/25 °C, day/night [33]. Similarly, Jiang and Huang [32] reported that leaf water content and EL% of perennial ryegrass and tall fescue was not affected by exposing plants only to heat stress of 35/30 °C, day/night for 12 days. Maintenance of the physiological functions under heat stress can be ascribed to the cooling effect of plants due to increased transpiration rate when there is enough soil moisture available. For instance, Jiang and Huang [32] observed transient increase of transpiration rates of perennial ryegrass and tall fescue plants during the first nine days of the heat stress compared to control plants. In the present study, canopy temperature depression of irrigated plants under high temperature treatments were often positive indicating that the canopies were cooler than surrounding air temperatures. Canopy temperatures at the control treatment were closer to air, but canopies were about 1.5 °C cooler under moderate heat stress and about 2 °C cooler under severe heat stress treatments. Chicory maintained the coolest canopies (3 °C cooler than air) under severe heat stress on day two of the severe heat stress treatment. Effective cooling of chicory relative to the grasses may be the reason for the lower reduction of leaf elongation rates of chicory during the second severe heat stress than grasses. 

#### 3.1.2. Effect of Drought Stress

In this study, leaf elongation rates of all pasture species decreased soon after drought stress was imposed and was below 0.5 cmd^−1^ at the end of both consecutive droughts (Figure 5d). For all the species, this pattern of decrease was more closely related to the pattern of RSWC (Figure 1d) rather than RLWC. This observation coincides with the results of Volaire and Lelièvre [11] who found a linear relationship between leaf extension and the fraction of soil water reserve available for plants. Michelena and Boyer [51] and Van Volkenburgh and Boyer [52] have also observed that the leaf elongation rates of maize decreased with increasing soil moisture deficit while there was no change in the turgor pressure in the growing points of leaves. Likewise, Meyer et al. [53] related the reduction of soybean hypocotyl growth rate from 1.6 to 0.2 mmh^−1^ with decline in soil water rather than hypocotyl turgor pressure. Since expansion of cells is not only related to turgor pressure but also to the supply of water to the growing area [54], declining soil water content could be the likely reason for the observed decline in leaf elongation rates of pasture species. 

In this study, cocksfoot maintained relatively higher leaf water content compared to other species during consecutive drought stresses. This may be related to better water uptake of cocksfoot at low soil moisture levels and delay of dehydration. In a comparison study between tall fescue and cocksfoot, Volaire and Lelièvre [11] found that leaf area and water potential of tall fescue decreased earlier than cocksfoot along with the increased cell membrane damage, indicating that cocksfoot possess higher dehydration control under drought stress than tall fescue. 

Other physiological parameters like Fv/Fm and EL% were not affected significantly during drought stresses in the tested species. Many other studies also agreed with these results indicating that short-term moderate water deficit had no effect on Fv/Fm [18] and cell membrane stability [55].

### 3.2. Effect of Combined Stresses and Acclimation to Previous Stress

Physiological parameters that were not affected by either heat and drought stress alone were severely affected when plants were exposed to combined heat and drought. Fv/Fm values for plants exposed to the first combined heat and drought treatments (at both moderate and severe temperatures) decreased below the values for non-stressed plants (0.83) suggesting physiological impairments of photosystem II, particularly in tall fescue and chicory. This pattern was consistent in the EL% and the RLWC in these two species; however, perennial ryegrass and cocksfoot were less affected during the first heat and drought stresses. Previous studies have shown that tall fescue has superior heat and drought tolerant capacity than perennial ryegrass [32], which is mainly related to a dehydration avoidance mechanism, from its efficient uptake of water from the deeper soil layers by the deep root system [56,57,58]. However, in this study, the roots of all the grass species reached the full length >75cm of the growing tubes and there was no difference of root dry weights between perennial ryegrass and tall fescue in moderate and severe heat and drought stress treatments. This result strongly suggests that under similar rooting conditions, tall fescue has no superior heat and drought tolerance compared to perennial ryegrass. Confirming this result, Wallner et al. [59] also found that tall fescue was no more heat tolerant than perennial ryegrass under in vitro conditions as assessed by cell membrane thermostability. In another study conducted in shallow pots (with restricted root growth), Milbau et al. [46] reported a reduction of Fv/Fm in both perennial ryegrass and tall fescue to around 0.2 after seven days of a heat wave of 35.8 ± 3.8 °C, in combination with drought stress, indicating no difference between tall fescue and perennial ryegrass in stress tolerance. Further, plants used in this study were raised from seeds and they were only eight weeks old when the treatments were imposed. Plants used in a similar study which resulted no substantial reduction Fv/Fm in chicory throughout the experiment, were about 8 months old [33] and those used by Jiang and Huang [32], who observed tall fescue perform better than perennial ryegrass, were 4 year old sod pieces.

In comparison with the first treatment, the second heat (both moderate and severe) and drought treatment affected pasture species differently. During the second moderate heat and drought stress treatment, Fv/Fm, EL% and RLWC were less affected in all species compared to the first treatment. This is likely to be due to the stress acclimation of plants. Many studies have demonstrated that plants can adapt to environmental stresses like heat and drought by modifying their membrane structure and functions and the production of heat stress proteins [41,60,61]. It has been shown that previous stress memory not only protects plants from subsequent stresses but also allows better performances [62,63]. The results of the current study are supported by Xu et al. [41], who reported that heat acclimation pretreatment (30 °C for three days) alleviated cell membrane damage and water loss from perennial ryegrass and tall fescue plants exposed to 38 °C.

Unlike in the second moderate heat and drought stress treatment, the second severe heat and drought treatment had detrimental impacts on all pasture species except chicory. During this time period, Fv/Fm values of all grass species decreased by 90% of the control, electrolyte leakage exceeded 90% and RLWC decreased below 20%, and these parameters only partially recovered at the end of the recovery period. In contrast, chicory’s tolerance of consecutive combined heat and drought stresses may be due to the maintenance of viable green leaf area. This is evidenced by high relative water content (>0.7) and high Fv/Fm values closer to 0.83 indicating that chicory can produce new biomass with the remaining viable new leaf area produced after the first stress treatment and supply photo assimilates to replenish diminishing carbohydrate reserves during the second stress. 

These results demonstrate that the plant species used in this study were better able to acclimate to consecutive moderate heat stress combined with drought stresses. This is an important characteristic in the cool season grasses to produce biomass under warmer and drier climates predicted for the coming decades in SE Australia. These results also suggest that tall fescue has similar stress tolerance compared to perennial ryegrass, which is in contrast with field studies that demonstrate that tall fescue is more persistent in pastures in south eastern Australia [64]. Greater persistence of tall fescue in field conditions might be due to its deeper roots and that this greater root depth is what confers greater survival under drier and hotter conditions [57,58]. The results of the current study support this by showing no difference between tall fescue and other grasses when the roots are the same length, suggesting that root depth is the key reason for its greater persistence under field conditions. This finding also highlights the importance of selecting or breeding deep-rooted species as an adaptation for the more variable future climates with frequent dry and hot conditions expected in southern Australia. Deeper rooting has been identified as a promising trait for drought adaptation in crops. For instance, in rice crops, cloning of *DEEPER ROOTING 1* (*DRO1*) gene to shallow rooting rice cultivar improved drought avoidance by modifying root system architecture through downward bending of roots [65]. In the current study, the likely reason for chicory’s superior heat and drought tolerance was its ability to maintain more viable and photosynthetically active leaf area however, other mechanisms relating to drought survival linked with root characteristics need further research. Further, species with Mediterranean origins may have different responses to combined heat and drought stresses and this is another future research direction. The current study focused only one cultivar from each species and in future research, the use of several genotypes with more than one cultivar would be helpful to screen more drought and heat tolerant species. Among all the species used in this study, chicory may be a potential summer active pasture species that is capable of tolerating more severe heat and drought stress and at the same time producing more livestock feed than grasses in SE Australia.

## 4. Materials and Methods 

### 4.1. Plant Materials and Establishment

The physiological responses of four summer active temperate pasture species including three grasses; perennial ryegrass (*Lolium perenne* cv. Base AR37), cocksfoot (*Dactylis glomerate* cv. Savvy), tall fescue (*Festuca arundinacea* cv. Quantum II Max P) and a herb; chicory (*Cichorium intybus* cv. Puna II) to heat and drought stress were evaluated in this study. All cultivars are commercially grown and popular among farmers in SE Australia. Quantum II max P is a commercial tall fescue cultivar bread in New Zealand (NZ) with higher summer production and drought tolerance [66]. Cocksfoot cv. savvy originated from continental Europe and is well known for drought tolerance and production under low soil fertility. Perennial ryegrass is less tolerant to drought than tall fescue and cocksfoot however, cv. base AR37 was bread in Australia from drought tolerant plants to enhance late season production. Perennial ryegrass is commercially grown in areas where annual rainfall is over 650 mm [67]. Chicory cv. puna II was selected from a commercial NZ line where it has higher summer production, drought resistance and persistence than grasses [68]. 

The experiment was conducted in the glass house and growth cabinet facilities at the Faculty of Veterinary and Agricultural Sciences, the University of Melbourne (37.7964°S, 144.9612°E). The experiment was conducted between 21 December 2017 and 3 April 2018. Plants were grown in polyvinyl chloride (PVC) tubes (diameter 10 cm, height 75 cm). A soil media consisting of a 3:1 (v/v) mixture of sand and soil. Soils (Red Chromosol) were collected from Dookie campus, the University of Melbourne. Before plant establishment, five poly vinyl chloride tubes were filled with the air-dried soil medium and then irrigated until free drainage occurred from the bottom of the tube and weighed (fully wet soil). These soils were oven dried at 80 °C for 10 days and dry weight was measured (oven dry soil), to be used later to calculate soil moisture content (described in Section 4.3). Plants were established by sowing six seeds in each tube. Plants were well watered daily to field capacity during the establishment period until the treatments commenced. Liquid fertilizer, aquasol (NPK 23:3.95:14) was applied every two days at seedling concentration (1 g/L:100 mL/plant) at the early stages of growth and increased gradually up to 5 g/L:100 ml/plant until the sixth week after sowing. At week six, osmocote (NPK 14:1.3:14.9 including iron magnesium and trace elements), a slow release fertilizer, was applied at a rate of 2 kg/m^3^ (nearly 12 g/pot). Plants were thinned out to retain three plants/pot at week six and a light grazing was simulated by clipping the plants to 2/3 height to encourage tiller growth. After eight weeks, plants were assigned to three separate growth chambers with 25 °C/15 °C day/night temperature, 12 h photoperiod, 900 µmolm^−2^s^−1^ light intensity and 70% RH for two weeks, to allow plants to adjust to the growth chamber conditions prior to imposing heat and drought treatments.

### 4.2. Treatments and Experimental Design

Heat and drought stress treatments commenced 70 days after sowing, following the acclimation period. Plants were exposed to three temperature regimes (control, moderate and severe heat stress) with or without watering as explained below. The stress levels were selected as moderate and severe at 30 °C and 35 °C, respectively, because the onset of heat stress occurs in the key pasture species (perennial ryegrass) at 30 °C and growth stops at 35 °C [50]. Treatments consisted of consecutive seven-day heat and drought stresses each followed by a seven-day recovery period. Conditions during the pretreatment and recovery periods were the same as the control. The heat stress treatments were designed to mimic the summer heat waves experienced in southern Victoria, Australia, where successive heat events are common [69]. Three heat treatments were imposed in separate growth cabinets as follows:

- Control: Plants were grown at 25 °C/15 °C day/night temperatures for the duration of the experiment.
- Moderate heat stress: Plants were grown at control conditions during pre-treatment week. A moderate heat stress was simulated by increasing the cabinet temperatures to 30 °C/20 °C day/night for a seven-day period followed by a seven-day recovery period (by resetting the growth chamber temperatures to the control conditions). The second seven-day heat stress was imposed in the same way followed by another seven-day recovery period.
- Severe heat stress: Two consecutive heat stresses and subsequent recovery periods were given in the same way to the moderate heat stress, but with increased temperatures (35 °C/25 °C day/night).

**Figure 9:**
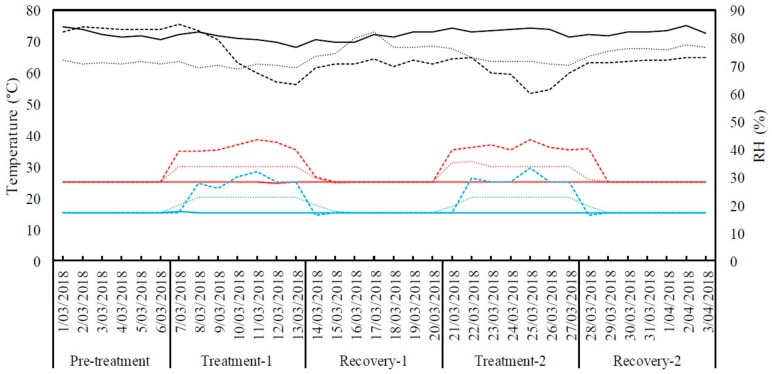
Daily maximum (red), minimum (blue) temperatures and average relative humidity (RH %) (black) inside the growth chambers for the duration of experiment. The thick line represents control (25 °C/15 °C day/night) treatment, the dotted line represents moderate heat stress treatment (30 °C/20 °C day/night) and the dashed line represents severe heat stress treatment (35 °C/25 °C day/night).

The diurnal pattern of the temperature variation was simulated in the growth chambers by changing the temperatures gradually between day and night. Environmental conditions of the three growth chambers during the experiment are shown in Figure 9.

Two watering treatments were applied in each growth chamber. One set of plants was irrigated each day to the field capacity for the entire duration (well-watered). Irrigation was withheld in another set of plants during the period of high temperature stress (drought). All plants were well-watered during the pre-treatment and the recovery periods.

The current study carried out inside growth chambers fairly resembles the natural environment in several ways. Pasture plants were grown in 75 cm long PVC tubes to allow more root growth into deep layers of the potting media. Further, diurnal variation of the temperature change was simulated by gradually increasing and decreasing the temperature between day and night. Finally, light intensity was maintained at 19.4 MJm^−2^ (~900 µmolm^−2^s^−1^) which is in the range of radiation intensity during heat waves in the SE Australia. For example, at Ellinbank, analysis of climate parameters indicated that the radiation intensity of days with a maximum daily temperature >30 °C reach on average 25 ± 5 MJm^−2^d^−1^ (data not shown).

There were 40 pots per growth chamber (two irrigation treatments × four species × five replicates) arranged in eight rows and five columns (blocking structure). Well-watered and drought stressed treatments were simulated in alternative rows of plants (eight in all). Five replicate plants of each species were randomly allocated in each watering treatment and the experimental design was randomized in row and column wise directions within each growth chamber. 

### 4.3. Measurements

Key physiological measurements such as maximum photochemical efficiency of PS II (Fv/Fm), cell membrane permeability (electrolyte leakage %), leaf elongation rate (cmd^−1^), leaf relative water content and the canopy temperature (°C) were measured at two to three-day intervals. Relative soil moisture content of the pots was also measured at one-day intervals. At the end of the experiment, plants were harvested and the dry weight of the canopy (green and senesced) (g), root length (cm) and root dry weight (g) were measured. 

Relative soil water content (RSWC) was calculated for each species to monitor soil dryness with time. Pots were weighed to measure the weight of soil with water. RSWC was calculated using Equation (1) [46].

(1) $$RSWC=\;\frac{Actual\;weight\;of\;water\;in\;tubes\;\left(Weight\;of\;soil\;with\;water-weight\;of\;oven\;dried\;soil\right)}{Potential\;weight\;of\;water\;in\;tubes\;\left(weight\;of\;fully\;wet\;soil-weight\;of\;oven\;dried\;soil\right)}$$

Chlorophyll fluorescence was measured using a MINI PAM (pulse amplitude modulation), portable chlorophyll fluorometer (Heinz Walz GmbH, Effeltrich, Germany). The maximum photochemical efficiency of PSII, Fv/Fm (Genty parameter) [70] was measured in dark-adapted leaves by selecting the youngest fully expanded leaves on the plant using the leaf clip holder 2030-B. The distance between the leaf and the fiberoptic was maintained at 15 mm during measurements. The angle between the fiberoptic axis and the leaf was 60°. Plants were subjected to darkness by turning lights off inside growth cabinets for one hour (in between 17.00–18.00) before taking measurements. Mode menu-5 (ML-BURST function) of the MINI PAM was used for the measurements to reduce the intensity of measuring light to 1/5 and clear any false signals in the fluorescence measurement. 

Cell membrane damage induced by heat and drought stresses was measured by testing the percentage electrolyte leakage (EL%) in leaves as described by Jiang and Huang [32]. Fully grown leaves were collected destructively from each pot and cut into small pieces of about 1 cm. Those leaf pieces were rinsed three times with distilled deionized water to wash out surface adhered electrolytes and those on the cut surfaces. Leaf pieces (5–10) were placed in test tubes and filled with 15 ml distilled deionized water. Test tubes were then shaken for 18 h in a mechanical shaker before measuring the initial conductivity (C_1_). Leaf samples were then autoclaved at 121 °C and 0.1 MPa for 15 minutes to kill the leaf tissues and the final conductivity (C_2_) was measured. EL% was computed as (C_1_/C_2_) × 100.

To calculate leaf elongation rates, a tiller with three fully expanded leaves and one emerging leaf was randomly selected from each pot and marked with a plastic wire ring at the base of the tiller. The length (cm) of the youngest elongating leaf was measured from tip to ligule of the youngest fully expanded leaf [71]. Leaf elongation rate was calculated by dividing the difference of the leaf length by the number of days and given as cmd^−1^. New tillers were selected every 2–4 days or when a new leaf started to emerge from the same tiller.

Relative leaf water content (RLWC) was measured according to the method used by Barrs and Weatherley [72] and Jiang and Huang [32]. First, the middle part of a fully-grown leaf was excised from each plant and fresh weight was recorded. Then the leaf was put into a water filled test tube and kept in the dark for five hours to reach full hydration. After five hours, the surface water was blotted dry using tissue paper and turgid weight was measured. Leaves were kept inside the oven at 60 °C for 48 h and dry weight was measured. RLWC was calculated using following equation.

(2) $$RLWC=\;\frac{Fresh\;Weight-Dry\;weight}{Turgid\;weight-Dry\;weight}$$

Canopy temperature was measured using infrared camera (FLIR T-series; model B 360) during the second, fourth and seventh day of the second treatment period. Thermal images of 3–5 plants in each species were captured using the white colored wall of the growth chamber as the background. The thermal images had a resolution of 320 × 240 pixels. Each pixel represents a specific temperature value of the picture. Thermal images were analyzed using a custom code developed using MATLAB R 2014b software [73]. Pixels from the background and the pot were excluded by selecting the maximum and minimum temperature value within the canopy and the pixels within the canopy were averaged to calculate canopy temperature. Air temperature inside growth chambers was measured using a mercury thermometer during the capture of each thermal image to get the accurate air temperature inside the cabinet. The difference between canopy temperature and air temperature (canopy temperature depression, CTD) was calculated as T canopy − T air.

At the end of the experiment, plants were harvested from the base near the soil surface and leaves of each plant were separated into green and senesced portions. Leaves were dried in the oven at 80 °C until constant weight achieved. Dry weight of the dead and green leaves was measured. 

Remaining soil column with roots was removed from the PVC tubes and roots were carefully washed to remove the trapped soil particles. Root system length was measured in each plant and roots were oven dried at 80 °C until a constant weight achieved. Dry weight of the roots was measured.

### 4.4. Statistical Analysis 

Data were statistically analyzed using linear mixed models, using GenStat statistical software. Rows and columns in each chamber were used as random effects in the model, while temperature, water status (irrigated or drought stressed) and species were used as fixed effects. Mixed models were used in this analysis because it could account for the lack of balance in the experimental design. Physiological measurements on each day were analyzed to reveal the differences between temperature, water and species and their interactions. 

## 5. Conclusions

This study demonstrated that temperate cool season pasture species can acclimate to combined moderate heat and drought stresses, but chicory is the only species that maintained growth under combined severe heat and drought stress. The results further showed that tall fescue has no superior drought and heat tolerance compared to perennial ryegrass under similar rooting conditions. Therefore, the ability of tall fescue to tolerate drought stress under field conditions may be largely due to its deep rooting and effective water uptake from the deeper soil layers. Individual stresses had no significant impacts on physiological functionality of pasture species, but the leaf elongation rates were affected by either stress alone. Irrigation mitigated the negative impacts of heat stress by cooling the pasture canopies 1–3 °C through transpirational cooling, with chicory having the coolest canopy temperature. While the specific mechanisms of chicory’s greater heat and drought tolerance compared to grasses deserve further research, it can be concluded that chicory is a potential pasture species to provide livestock feed under more challenging hot and dry summer months in SE Australia. 

## Figures and Tables

**Table 1:** Level of significance of the main factors and their interactions in relative soil water content.

Main Factors and Interactions	Pre-Treatment			Treatment 1			Recovery 1			Treatment 2			Recovery 2		
	Day 1	Day 3	Day 6	Day 2	Day 4	Day 7	Day 3	Day 5	Day 7	Day 2	Day 4	Day 7	Day 2	Day 5	Day 7
Temperature	NS	NS	NS	NS	NS	NS	NS	NS	NS	NS	NS	NS	NS	NS	NS
Water status	NS	NS	NS	***	***	***	*	NS	NS	***	***	***	*	NS	*
Species	*	***	***	***	***	*	NS	NS	NS	*	NS	*	*	NS	NS
Temperature × Water status	NS	NS	NS	**	***	NS	NS	NS	NS	NS	**	NS	NS	NS	NS
Temperature × Species	NS	NS	**	**	**	NS	NS	NS	NS	NS	NS	NS	NS	NS	NS
Water status × Species	NS	NS	NS	**	***	NS	*	*	NS	*	*	NS	*	NS	*
Temperature × Water status × Species	NS	NS	NS	NS	*	*	NS	NS	NS	NS	*	*	NS	NS	NS

NS = Non-significance, * *p* < 0.05, ** *p* < 0.005 and *** *p* < 0.001.

**Table 2:** Level of significance of the main factors and their interactions on each day for maximum photochemical efficiency of photosystem II (Fv/Fm).

Main Factors and Interactions	Pre-Treatment	Treatment 1			Recovery 1			Treatment 2			Recovery 2		
	Day 0	Day 1	Day 3	Day 6	Day 2	Day 4	Day 6	Day 1	Day 3	Day 6	Day 1	Day 4	Day 6
Temperature	NS	NS	NS	NS	NS	NS	NS	NS	NS	NS	NS	NS	NS
Water status	NS	NS	NS	**	***	***	NS	NS	*	***	***	***	***
Species	***	***	***	*	***	NS	***	*	***	***	***	***	***
Temperature × Water status	NS	NS	*	*	***	*	NS	NS	*	***	NS	***	***
Temperature × Species	NS	NS	NS	**	***	*	NS	NS	NS	***	*	***	***
Water status × Species	NS	NS	NS	***	***	*	NS	NS	NS	**	NS	***	***
Temperature × Water status × Species	NS	NS	NS	**	***	NS	NS	NS	NS	**	NS	***	***

NS = Non-significance, * *p* < 0.05, ** *p* < 0.005 and *** *p* < 0.001.

**Table 3:** Level of significance of the main factors and their interactions on each day for cell membrane permeability.

Main Factors and Interactions	Pre-Treatment	Treatment 1		Recovery 1		Treatment 2		Recovery 2	
	Day 0	Day 3	Day 6	Day 2	Day 6	Day 3	Day 6	Day 3	Day 6
Temperature	NS	NS	NS	NS	NS	NS	NS	***	NS
Water status	NS	NS	***	***	**	*	***	***	***
Species	NS	***	***	***	***	***	***	***	NS
Temperature × Water status	NS	NS	***	***	NS	NS	***	***	***
Temperature × Species	NS	NS	***	***	NS	NS	***	***	***
Water status × Species	NS	NS	***	***	NS	NS	***	***	***
Temperature × Water status × Species	NS	NS	***	***	NS	NS	***	***	***

NS = Non-significance, * *p* < 0.05, ** *p* < 0.005 and *** *p* < 0.001.

**Table 4:** Level of significance of the main factors and their interactions on each day for leaf elongation rate.

Main Factors and Interactions	Pre-Treatment		Treatment 1			Recovery 1			Treatment 2			Recovery 2		
	Day 3	Day 6	Day 2	Day 4	Day 7	Day 3	Day 5	Day 7	Day 2	Day 4	Day 7	Day 2	Day 5	Day 7
Temperature	NS	NS	NS	NS	NS	NS	NS	NS	NS	NS	NS	NS	NS	NS
Water status	NS	NS	*	***	***	***	NS	NS	NS	***	***	***	***	***
Species	***	***	***	***	***	NS	**	*	***	***	***	***	*	***
Temperature × Water status	NS	NS	NS	NS	NS	NS	NS	NS	NS	NS	*	NS	NS	NS
Temperature × Species	NS	NS	NS	NS	*	NS	NS	NS	NS	NS	NS	*	NS	NS
Water status × Species	NS	NS	NS	NS	***	***	NS	***	***	NS	NS	NS	*	*
Temperature × Water status × Species	NS	NS	NS	NS	***	NS	NS	NS	NS	NS	NS	NS	NS	NS

NS = Non-significance, * *p* < 0.05, ** *p* < 0.005 and *** *p* < 0.001.

**Table 5:** Level of significance of the main factors and their interactions on each day for relative leaf water content.

Main Factors and Interactions	Pre-Treatment	Treatment 1		Recovery 1		Treatment 2		Recovery 2	
	Day 0	Day 3	Day 6	Day 2	Day 6	Day 3	Day 6	Day 3	Day 6
Temperature	NS	NS	NS	NS	NS	NS	NS	NS	NS
Water status	NS	*	***	***	*	***	***	***	***
Species	***	***	*	***	***	NS	***	*	NS
Temperature × Water status	NS	NS	NS	*	NS	NS	***	***	***
Temperature × Species	NS	NS	NS	NS	NS	NS	***	***	NS
Water status × Species	NS	NS	NS	***	NS	*	***	***	***
Temperature × Water status × Species	NS	NS	NS	NS	NS	NS	***	**	*

NS = Non-significance, * *p* < 0.05, ** *p* < 0.005 and *** *p* < 0.001.

**Table 6:** Level of significance of canopy temperature depression measured on day 2, 4 and 7 in the second treatment period.

Main Factors and Interactions	Treatment 2		
	Day 2	Day 4	Day 7
Temperature	NS	NS	NS
Water status	***	***	***
Species	***	*	NS
Temperature × Water status	NS	NS	***
Temperature × Species	NS	NS	NS
Water status × Species	NS	NS	NS
Temperature × Water status × Species	NS	NS	NS

NS = Non-significance, * *p* < 0.05, ** *p* < 0.005 and *** *p* < 0.001.

**Table 7:** Level of significance of the top weight (green), top weight (senesced), root weight and root length measured at the end of the experiment.

Main Factors and Interactions Terms	Top Dry Weight (Green)	Top Dry Weight (Dead)	Root Dry Weight	Root Length
Temperature	NS	NS	NS	NS
Water status	***	***	***	**
Species	***	*	NS	***
Temperature × Water status	***	***	NS	NS
Temperature × Species	*	***	NS	*
Water status × Species	**	*	**	NS
Temperature × Water status × Species	NS	***	NS	NS

NS = Non-significance, * *p* < 0.05, ** *p* < 0.005 and *** *p* < 0.001.
